# Patient preferences for future care - how can Advance Care Planning become embedded into dementia care: a study protocol

**DOI:** 10.1186/1471-2318-10-2

**Published:** 2010-01-12

**Authors:** Louise Robinson, Claire Bamford, Fiona Beyer, Alexa Clark, Claire Dickinson, Charlotte Emmet, Catherine Exley, Julian Hughes, Lesley Robson, Nikki Rousseau

**Affiliations:** 1 Institute of Health and Society, Newcastle University, Newcastle upon Tyne, NE2 4AA, UK; 2 Newcastle upon Tyne Primary Care Trust, Newcastle upon Tyne, UK; 3 Northumbria University, Newcastle upon Tyne, UK; 4 North Tyneside General Hospital, Northumbria Healthcare NHS Foundation Trust*, UK

## Abstract

**Background:**

People living with a long term condition may wish to be able to plan ahead, so that if in future they cannot make decisions, their wishes about their care will be known; this process is termed Advance Care Planning (ACP). In dementia, guidance stipulates that ACP discussions should take place whilst the person still has capacity to make decisions. However there is a lack of evidence on the effectiveness of ACP in influencing patient choice and resource use. The aims of this study are to determine the effectiveness of ACP in dementia care, identify the factors which facilitate the process in practice and provide a better understanding of the views and experiences of key stakeholders in order to inform clinical practice.

**Methods/Design:**

The four phase project comprises a systematic review (Phase 1) and a series of qualitative studies (Phases 2 and 3), with data collection via focus groups and individual interviews with relevant stakeholders including people with dementia and their carers, health and social care professionals and representatives from voluntary organisations and the legal profession. The conduct of the systematic review will follow current best practice guidance. In phases 2 and 3, focus groups will be employed to seek the perspectives of the professionals; individual interviews will be carried out with people with dementia and their carers. Data from Phases 1, 2 and 3 will be synthesised in a series of team workshops to develop draft guidance and educational tools for implementing ACP in practice (Phase 4).

**Discussion:**

In the UK, there is little published research on the effectiveness of ACP, despite its introduction into policy. This study was designed to explore in greater depth how ACP can best be carried out in routine practice. It affords the opportunity to develop both a theoretical and practical understanding of an area which both patients and professionals may find emotionally challenging. Importantly the study will also develop practical tools, which are grounded in practice, for all relevant stakeholders to enable the facilitation of timely and sensitive ACP discussions.

## Background

Life expectancy is increasing by two years every decade. Currently older people represent the fastest growing sector of our population, with those over 60 years of age comprising one fifth of the population. The largest population increase will be seen in the *oldest old *i.e. people over 85 years. An ageing population will undoubtedly lead to an increased incidence of long term conditions, especially age-related illness such as osteoarthritis and dementia [1].

In the United Kingdom, improving the health and social care of our ageing population is one of the government's strategic priority areas [2,3]. However there is clear evidence that the quality of care received by some older people is suboptimal, for example people with advanced dementia experience inappropriate hospital admissions, inadequate symptom management and a lack of integrated care at the end of life [4,5]. In addition in terms of patient choice, most older people would choose to die in their own homes [6,7]; however very few people with dementia die at home or in a hospice [8].

People living with a long term condition, especially those with dementia, may wish to be able to plan ahead, so that if in future they cannot make decisions, their wishes about their care will be known; this process is termed Advance Care Planning (ACP). Advance directives (ADs) or living wills are documents in which an adult can record preferences for future care. ADs first appeared in the United States (US) in the 1960s and became more common after 1990, when the US government introduced the Patient Self Determination Act which requires Medicare and Medicaid providers to inform patients of their right to complete an advance directive, at the time of admission. In addition to an "advance directive or decision" stating informed consent to refuse specific treatment if loss of mental capacity ensues, people can nominate others to make decisions on their behalf, should they lose mental capacity, in the areas of finance and/or health and personal welfare (Lasting Power of Attorney). The legal framework regarding loss of capacity/competency can vary between countries, and in the US, between states.

In terms of the processes that facilitate AD completion rate, systematic reviews have concluded that multi-component interventions, particularly one to one discussions with a skilled facilitator over a period of time, are the most effective [9-14]. However, even with such interventions, ADs may not be adequately disseminated to influence patient care and health service use [12]. In dementia care, guidance stipulates that such care planning discussions should take place whilst the person still has capacity to make decisions [15]. The number of ADs made by people with dementia has traditionally been lower than for people with cancer [16]. In terms of the impact of ACP on dementia care, a reduction in unnecessary hospital admissions after ADs has been shown [17,18]. A Cochrane review is currently underway to assess the effects of interventions to promote the use of ADs in end of life decision-making in all adults [19]. However there is a lack of evidence on the effectiveness ACP in areas where loss of capacity will undoubtedly occur such as in dementia and also a lack of understanding as to why people with dementia are less likely than those with cancer to complete an AD. Advance care planning may be particularly relevant in conditions such as dementia, where patients are likely to experience an extended period of mental incapacity but may still be in relatively good physical health, so that ACP may need to consider aspects of care beyond the end-of-life decisions that are the focus of most advance directives.

In the United Kingdom (UK), the End of Life Care Strategy was introduced to ensure that people's individual needs, priorities and preferences for end of life care were identified, documented, reviewed, respected and acted upon wherever possible [20]. Although guidance on ACP is available for both professionals and patients [1,21,22], the process does not yet appear to be embedded into routine practice [23] and factors which facilitate the process need to be defined. Research is urgently needed to address both the knowledge transfer gap to facilitate the integration of ACP into routine clinical practice and to establish whether ADs, if implemented, influence patient care and choice [12,23,24].

This paper describes the protocol of a multidisciplinary study that will provide timely and essential insights into an area of end of life care, ACP, which has become policy driven in the UK despite a weak evidence base. The project will particularly focus on ACP in dementia where people are highly likely to experience prolonged periods of mental incapacity during which it will be important for health professionals to know an individual's preferences for care. However it will also determine the factors that, in general, facilitate or hinder people taking part in such potentially sensitive discussions, in order to provide a better understanding of how the process of ACP can become better integrated into the routine care of people with long term conditions and in particular those living with dementia.

## Methods/Design

The aim of this study is to explore how the process of ACP can become implemented in the routine care of people with long term conditions, and in particular dementia, where loss of capacity will inevitably occur and where communication and memory difficulties may make such ACP discussions more challenging. The study will provide a better understanding of the views and experiences of all relevant stakeholders, including patients, family carers and professionals, in order to inform clinical practice. Specifically it will:

1. Define the factors which facilitate and inhibit the process of ACP in practice;

2. Explore how ACP can be implemented in potentially more challenging situations such as in dementia care;

3. Critically appraise and synthesise existing evidence on the effectiveness of ACP in dementia;

4. Identify the professional competencies and training required to implement ACP;

5. Develop practical guidance for professionals, patients and carers on ACP with a focus on dementia care.

The project comprises a systematic review and a series of qualitative studies, with data collection via focus groups and individual interviews. Focus groups will be employed to seek the perspectives of health and social care professionals, supplemented with interviews to capture the views of professional groups not represented in the focus groups. Focus groups enable the researcher to capitalise on communication and critical discussion between research participants in order to generate data; it is often recommended that focus groups be homogenous but the advantages of including a more diverse group are also recognised (i.e. a range of professions). Individual interviews will be used to explore the perspectives of people living with a long term condition such as those with dementia, and their family carers on ACP. Focused interviews are a particularly useful tool to employ in an area where little is known, they are also flexible enough to allow the interviewer and interviewee to explore potentially sensitive and challenging issues which are pertinent to the individual.

### Phase 1: Existing evidence on the effectiveness and feasibility of ACP

The aims of Phase 1 are:

• To determine the factors which influence the implementation of ACP, to allow us to learn from and build upon experiences of ACP in other populations and settings.

• To assess the effectiveness and acceptability of ACP in one specific long term illness, dementia.

#### Methods

In order to refine the scope of our review and identify existing reviews, preliminary pilot searches were carried out. Key databases were searched including but not limited to Medline, Embase, Cinahl, the Cochrane Database of Systematic Reviews (CDSR) and Sociological Abstracts. This identified a number of systematic reviews which focused on the effectiveness of interventions to promote the use of ACP in a variety of health care settings [9-14]. These reviews included almost exclusively quantitative sources of evidence about ACP. In addition, most focused on the effectiveness of different types of intervention to facilitate the completion of ADs; there were few which explored the effectiveness of ADs on influencing patient outcomes, such as preferred place of care, and health resource use e.g. unplanned hospital admissions [10,12].

Also identified was a recent protocol registered with the Cochrane Collaboration on *Interventions for promoting the use of advance directives for end of life decisions in adults *[19]. This systematic review will appraise and synthesise evidence on AD research in populations including all adults aged over 18 with no restrictions on their health status. On completion of the pilot searches we concluded that a systematic review focused specifically on the effectiveness of ACP in an area of long term care where loss of capacity was inevitable, and where such discussions may be more challenging as in dementia, is warranted. The conduct of the systematic review in terms of identifying and synthesising relevant quantitative data will follow current best practice guidance [25].

### Phase 2: Health professionals' experiences of ACP in practice

The aims of Phase 2 are:

• To explore health professionals' views and experiences of carrying out ACP.

• To determine the factors which facilitate or inhibit the implementation of ACP.

• To identify the professional competencies essential for carrying out ACP in practice.

#### Sampling

The sample (up to 60) will include health care professionals from one Primary Care Trust (PCT) in the UK. As part of the NHS End of Life Programme, Newcastle upon Tyne PCT Specialist Palliative Care Team has developed training on ACP and identified documentation for staff to use from a review of existing documents [20]. The ACP process is facilitated by healthcare professionals, who have received the appropriate training and is for any patient defined as having a life-limiting illness and at risk of dying within 1 year; within primary care, these will be mainly patients on the practice palliative care register. Training was introduced in 2007 and to date, 85 health care professionals from the trust have received the training. A purposive sample of health care professionals will be selected to reflect:

i) a range of clinical perspectives including General Practitioners (GPs), community nurses and multidisciplinary members of community rehabilitation teams and

ii) a range of experience of ACP, i.e. those who have received training, those who have indicated a desire to undertake the training but not yet received it, and those who have been offered ACP training but declined to participate.

#### Methods

Focus groups (up to 6), and individual interviews (up to 20) for those unable to attend the focus group, will be conducted. Where possible, the focus groups will include participants of similar experience and professional background. The focus groups/interviews will explore:- participant views and experiences of carrying out ACP in practice, including both the process and documentation; the factors that promote or inhibit ACP in clinical practice; the skills and competencies required to carry out ACP successfully and whether any extra training or resources are needed, and any areas where they feel ACP is more difficult to carry out in practice. Potential participants will be initially contacted by Newcastle upon Tyne PCT Community Palliative Care Team to inform them about the study; those expressing interest in the study will be sent a letter from the project team and a study information sheet. Potential participants will have up to 1 week to decide if they wish to be involved. Written consent will be secured prior to data collection.

### Phase 3: ACP in dementia care

#### Aim

The aims of phase 3 are

• To explore the views and experiences of key stakeholders (people with dementia, their carers and relevant professionals) on the process and timing of ACP in dementia care.

• To determine the factors which facilitate or inhibit the implementation of ACP in dementia care.

• To identify the professional competencies essential for carrying out ACP in dementia care.

#### Sample

Up to twenty people with mild dementia (Mini Mental State Examination score ≥21) and their main lay carer (up to 20); a purposive sample will be sought to ensure maximum variation of age, type of carer and living situation. Potential participants will be identified through Old Age Psychiatry Services in 2 secondary care trusts. People with dementia will initially be contacted by a professional known to them to ascertain if they are willing to be approached about the study. If they are, a letter and study information sheet will be sent from the project team. They will have 1 week to decide if they wish to take part. Written consent would be secured prior to the interview beginning; if this were not possible then verbal consent would be recorded. Patients would be reassured that declining to participate, or subsequently withdrawing from participation, would not affect their care.

Sampling in this phase will also include representatives from the following professional groups: general practitioners (GPs), community nurses, old age psychiatrists, community psychiatric nurses (CPNs), specialists in the care of younger people with dementia, psychologists, social care professionals, legal professionals and advocacy groups e.g. Alzheimer's Society. Health care professionals will be recruited from the participating trusts; social care professionals will be recruited from Social Services and advocacy representatives from advocacy groups (e.g. Alzheimer's Society; Age Concern) within the study area. Legal representatives will be identified from either advocacy groups or university departments of law (Newcastle/Northumbria). A letter and study information sheet would be sent from the project team. Potential participants will have 1 week to decide if they wish to become involved. Written consent would be secured prior to data collection.

#### Methods

Interviews will be used to gather the views of people with dementia and their carers. Specifically, we shall enquire about their views on ACP discussions, how they feel about taking part in such discussions, who they might involve, which aspects of ACP they feel are most important, and their views on the timing of ACP and current ACP documents in use (such as the form used by participating professionals in WP2 and examples identified from WP1). We would also ask whether they have any ACP plans in place already.

Focus groups will be used to gather data from the professionals. We will explore participant views and experiences of carrying out ACP in dementia care in practice, including both the process, timing and documentation used; the factors that promote or inhibit ACP; the skills and competencies required to carry out ACP successfully and whether any extra training or resources are needed and which professionals are best placed to discuss and document ACP in dementia care.

### Data preparation and analysis for Phases 2 and 3

All focus groups and interviews will be digitally recorded and transcribed verbatim. In line with Data Protection Legislation and Research Governance, all information pertaining to individuals would be anonymised. Following the principles of the 'constant comparative method' [26], data collection and analysis will occur concurrently to allow for issues from earlier interviews to be explored in more depth in subsequent interviews. Thematic analysis will be conducted on the transcripts. The validity of data interpretation will be ensured by independent coding and cross-checking by at least two members of the research team. The software package NVivo will be used to facilitate data management. Regular data analysis meetings will be held throughout the data collection and analysis period by the project team which includes representatives from primary and secondary care, social science, social work and psychology.

### Phase 4: ACP in health care: guidance for best practice

The aims of phase 4 are:

• To develop guidance for patients, carers and health and social care professionals on how best to implement ACP in practice.

• To create educational tools for health and social care professionals to enable the facilitation of timely and sensitive ACP discussions with people with long term conditions and in particular those living with dementia.

The project team will synthesise data from Phases 1, 2 and 3 in a series of team workshops to develop draft guidance and educational tools for implementing ACP in practice. The guidance will also identify who is best placed to facilitate this process and the skills and competencies required. We will also seek the opinions of our study participants, including legal, health and social care professionals and people with dementia and their carers, on our initial draft guidance. Two task groups will be held (up to 8 people in each) with participants from phase 3, comprising i) legal, health and social care participants and ii) people with mild dementia and their carers. The proposed guidance and educational tools will also be presented to the project external advisory group for feedback. Following analysis of data from these groups, the guidance and tools will be refined by the project team before further dissemation.

### Ethical approval

This study has approval from Newcastle and North Tyneside 1 Research Committee (09/H0906/5) and research governance approval from each participating trust site. Members of the project team in direct contact with patients have been issued with NHS honorary contracts.

## Discussion

Following our initial pilot searches, we identified a significant number of recent systematic reviews, albeit largely US-based, which mainly synthesised quantitative data on the effectiveness of different interventions to influence ACP outcomes. These reviews largely included evidence from North America and reflected a particular setting where legislation i.e. the Patient Self Determination Act, has influenced ACP; such findings may not be relevant to other countries. Although we also discovered a large number of qualitative studies on aspects of ACP in a variety of health care settings, a review or synthesis of this evidence does not appear to have been undertaken. Such a synthesis of the wider qualitative research literature on ACP in all health care settings, in order to identify the factors which influence implementation of ACP discussions in any setting, may be a useful addition to existing evidence. However there is currently a lack of consensus as to how best to undertake a systematic review of qualitative literature, although guidance on the process of searching for qualitative evidence [27,28] and approaches to qualitative data synthesis in a systematic review are available [29-34].

Prior to the start of this study, there was very little published UK research on the effectiveness of ACP, despite its introduction into NHS policy on End of life Care, although research is beginning to emerge [23]. This study was designed to address gaps in the literature firstly by evaluating both the effectiveness and feasibility of ACP in one particular area of long term care, dementia where loss of capacity is inevitable, but also through exploring in greater depth how ACP can best be carried out in routine practice. This study affords the opportunity to develop both a theoretical and practical understanding of an area which both patients and professionals may find emotionally challenging to undertake. Importantly, the study will also develop practical tools, which are grounded in practice, for all relevant stakeholders to enable the facilitation of timely and sensitive ACP discussions.

## Competing interests

The authors declare that they have no competing interests.

## Authors' contributions

LR, CB and CE conceived the study; LR, CB, CE, AC, LesR and JH were all involved in the original design of the study. FB designed the search strategy for the review. NR and CD are involved in the design and conduct of data collection at all stages. LR drafted this paper. All authors were involved in revising the manuscript and have given approval of the final manuscript.

## Pre-publication history

The pre-publication history for this paper can be accessed here:

http://www.biomedcentral.com/1471-2318/10/2/prepub
